# The effect of cardiopulmonary bypass on blood thiamine concentration and its association with post-operative lactate concentration

**DOI:** 10.1186/s13019-022-02016-0

**Published:** 2022-10-07

**Authors:** Andrea L. Odelli, Adam Holyoak, Sumit Yadav, Sarah M. Page, Daniel Lindsay

**Affiliations:** 1 Intensive Care Unit, John Hunter Hospital, Lookout Road, New Lambton Heights, Newcastle, NSW 2305 Australia; 2 Medical Superintendent, Thursday Island Hospital, Thursday Island, QLD Australia; 3 Director of Cardiothoracic Surgery, Townsville University Hospital, Douglas, QLD Australia; 4 Department of Cardiothoracic Surgery, Gold Coast University Hospital, Gold Coast, QLD Australia; 5 James Cook University, Douglas, QLD Australia

**Keywords:** Thiamine, Cardiac surgery, Cardiopulmonary bypass, Extracorporeal circulation

## Abstract

**Objective:**

Cardiothoracic surgery is a large field in Australia, and evidence suggests post-cardiopulmonary bypass (CPB) hyperlactataemia is associated with higher morbidity and mortality. Low thiamine levels are a potentially common yet treatable cause of hyperlactataemia and may occur in the setting of exposure to CPB non-biological material. We hypothesized that cardiopulmonary bypass would result in decreased whole-blood thiamine levels, which may therefore result in increased whole-blood lactate levels in the post-operative period.

**Methods:**

Adult patients undergoing non-emergent CPB were recruited in a single centre, prospective, analytic observational study at Townsville University Hospital, Australia. The primary outcome was a comparison of pre- and post-CPB thiamine diphosphate level, secondarily aiming to assess any relationship between lactate and thiamine levels. Prospective pre- and post-CPB blood samples were taken and analysed at a central reference laboratory.

**Results:**

Data was available for analysis on 78 patients. There was a statistically significant increase in thiamine diphosphate level from pre-CPB: 1.36 nmol/g Hb, standard deviation (SD) 0.31, 95% confidence intervals (CI) 1.29–1.43, to post-CPB: 1.77 nmol/g Hb, SD 0.53, 95% CI 1.43–1.88, *p* value < 0.001. There was a non-statistically significant (*p* > 0.05) trend in rising whole-blood lactate levels with increasing time. Analysis of lactate levels at varying time periods found a significant difference between baseline measurements and increased levels at 13–16 h (*p* < 0.05). There was no significant relationship observed between whole-blood thiamine levels and post-operative lactate levels.

**Conclusion:**

Whole-blood thiamine levels were found to increase immediately post-CPB in those undergoing elective cardiac surgery. There was no correlation between whole-blood thiamine levels and post-operative arterial lactate levels.

## Background

Cardiothoracic surgery is common in Australia, with over 12,000 patients undergoing such procedures in 2015 [1]. Within this cohort, significantly higher morbidity and mortality is associated with the occurrence of post-operative hyperlactataemia following cardiopulmonary bypass (CPB) [1–3].

Lactate is produced as a byproduct of anaerobic metabolism when anoxia prevents pyruvate from entering the tricarboxylic acid cycle [4]. Hyperlactataemia is typically classified as type A, associated with tissue hypoxia or hypoperfusion, or type B in the absence of such features. Tissue hypoxia and hypoperfusion are often present in the post-operative cardiothoracic patient, however type B hyperlactataemia is frequently also present, albeit poorly understood [5]. Indeed, a challenging aspect to the analysis of hyperlactataemia outcomes within the cardiothoracic surgical cohort is that there is commonly co-existence of the two forms. For example, adrenaline (epinephrine) associated hyperlactatemia is thought to be due to accelerated aerobic metabolism and is not associated with the same negative adverse outcomes, however the utilization of adrenaline in the cardiothoracic surgical cohort likely implies a degree of impaired cardiac output, perfusion and oxygen delivery [4]. There is also a recognized phenomenon of late-onset type B hyperlactataemia, occurring approximately 6–12 h post-operatively, that is poorly understood though appears to be a benign process [6].

**Fig. 1:**
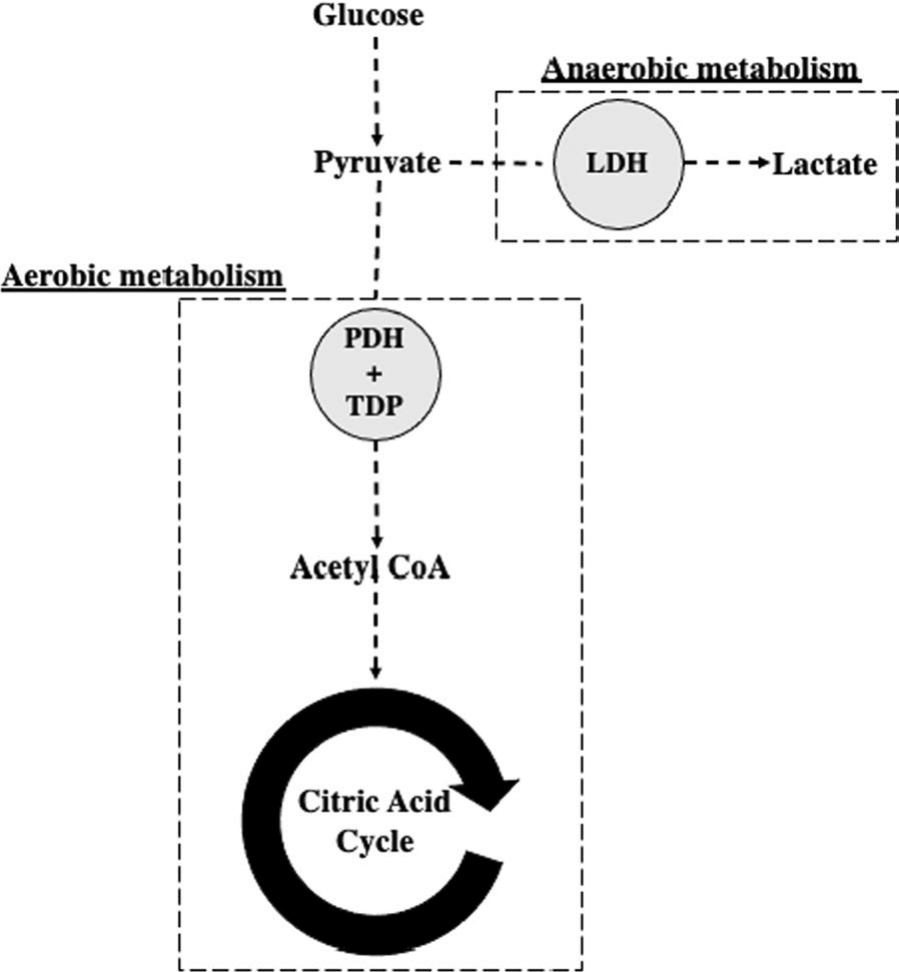
The role of thiamine diphosphate (TDP) as a co-factor for pyruvate dehydrogenase (PDH) in aerobic metabolism. LDH, lactate dehydrogenase; CoA, coenzyme A

Thiamine, in the form of thiamine diphosphate (TDP), is required for aerobic metabolism [7]. It is an essential cofactor for pyruvate dehydrogenase (PDH), the enzyme responsible for the conversion of pyruvate to acetyl coenzyme A, which subsequently enters the citric acid cycle (Fig. 1) [7]. Hence, thiamine depletion will redirect pyruvate to increase lactate production [8]. Low thiamine levels are a potentially common yet readily treatable cause of hyperlactataemia. We hypothesized that cardiopulmonary bypass would result in decreased whole-blood thiamine levels, which may therefore result in increased whole-blood lactate levels in the post-operative period. A cause of decreased thiamine levels in the setting of cardiac surgery may be exposure to non-biological membranes and tubing associated with extracorporeal circulation or altered PDH activity [9, 11].

Thiamine kinetics in patients undergoing cardiothoracic surgery is complex and poorly understood. There is a paucity of literature examining thiamine depletion in cardiothoracic surgery. Donnino et al. and Anderson et al. found a statistically significant decrease in thiamine levels from pre- to post-CPB in patients undergoing cardiothoracic surgery [10, 11]. Additionally, one study identified an inverse relationship between thiamine levels and lactate levels [11]. Based on these two studies demonstrating post-CPB thiamine depletion, multiple randomized control trials (RCT) have analysed the effects of thiamine administration on post-operative lactate levels in patients undergoing CPB, compared with placebo [12–14]. While no RCT has shown a statistically significant effect of thiamine supplementation on post-operative lactate level, studies performed to date have been under-powered to detect this effect [12–14].

Both studies demonstrating post-CPB thiamine depletion had small sample sizes that inhibited quantification of the likely incidence of this occurrence and limited conclusions on the influence of CPB itself [10, 11]. We have therefore conducted an investigation of pre- and post-CPB thiamine levels, and sought to examine whether a relationship exists between post-operative thiamine and lactate levels.

## Methods

### Study design

A single centre, prospective, analytic observational study was conducted at Townsville University Hospital, a tertiary public hospital and the major referral centre for North Queensland. The protocol was approved by the Townsville Hospital and Health Service Human Research Ethics Committee in December 2017 (HREC/17/QTHS/267). Written consent was obtained from all participants.

### Study population

Eighty-two patients receiving cardiac surgery involving cardiopulmonary bypass were recruited between May 2018 and October 2018. The study included all non-emergent cardiothoracic surgical patients undergoing cardiopulmonary bypass aged 18 years or older that were admitted to the intensive care unit (ICU) following surgery. Excluded were patients aged < 18 years, those that were pregnant, those receiving thiamine supplementation, those undergoing pre-operative dialysis, those undergoing emergency surgery or surgery not requiring CPB, and those who refused their consent.

### Data collection

The following data were extracted from the electronic patient medical record for each participant enrolled: age, gender, height, weight and body mass index (BMI), pre-operative haemoglobin and creatinine levels, the presence or absence of diabetes mellitus, type of surgery and surgery characteristics. An additional 4 ml whole blood ethylene-diamine-tetraacetic acid (EDTA) tube was drawn from the arterial catheter of each participant pre- and immediately post-operatively upon their arrival to ICU. The sample was first sent to TUH haematology department whereby appropriately trained and credentialed laboratory technicians utilized the automated analyser Sysmex XN3000 to measure a whole-blood haemoglobin (Hb) level. The EDTA sample was then frozen to − 70 °C and sent via courier with temperature monitoring and light protection to the Pathology Queensland laboratory located at the Royal Brisbane and Women’s Hospital, Queensland. The whole-blood TDP level was measured by a standard technique with reverse phase isocratic high performance liquid chromatography (HPLC) coupled with fluorescence detection [15]. Measurement was undertaken by appropriately trained and credentialed RBWH laboratory technicians in HPLC analytical chemistry. Equipment utilised was supplied by Chromsystems Germany and the assay performed on a Waters Alliance 2695 Separations module coupled to 2475 Fluorescence detectors (Waters Corporation, Milford, MA, USA). Sample preparation was a two-step procedure where whole blood was deproteinised and the extract derivatised before injecting onto the column. The concentration of TDP was initially measured as nanomoles per litre (nmol/L) then calculated and presented to two-decimal places as an Hb-ratio for each patient in nanomoles per gram of haemoglobin (nmol/g Hb), with normal reference range of 0.90–1.95 nmol/g Hb.

Post-operatively, 1–2 ml of blood was aspirated from the arterial catheter of each patient immediately on ICU admission and four-hourly thereafter until 24 h was reached or ICU discharge occurred. This protocol conformed with the established standard of care of post-operative cardiothoracic patients within TUH ICU. Whole-blood arterial samples were processed immediately by appropriately trained and credentialed ICU nurses on the ABL800flex blood gas analyser machine, a demonstrated reliable method of point-of-care lactate measurement [16].

### Outcomes

The primary outcome was a comparison of the pre- and post-CPB TDP level. The secondary outcome was an analysis of any correlation between thiamine and blood lactate levels.

### Sample size

An estimated required sample size of 40 patients was calculated using GPower 3.1.9.2 test (Wilcoxin Signed rank test) based on prior literature informing an estimated difference in pre- and post-CPB thiamine levels of 5.5 nmol/L, to give a 95% power and type 1 error of *p* = 0.05 [10, 11]. The estimated sample size was electively doubled with recruitment of a total of 80 subjects, given the real difference in thiamine levels is largely unknown, thus improving the likelihood of a valid result if estimates of change were inaccurate.

### Statistical analysis

Study population demographics were analysed and presented using descriptive statistics, with the mean and data range displayed. A statistician was consulted for analysis of results and utilized RStudio version 1.2. Pre- and post-operative whole-blood thiamine levels were assessed and compared using paired samples t-test, provided normality of data distribution, and presented as means with standard deviations (SD). Post-operative whole-blood lactate levels were assessed by the Kruskal–Wallis test to assess for differences between time-points. Repeated measures analysis of variance was utilised for comparison of multiple lactate measurements at different time points. Regression analysis was performed by utilising the Spearman’s rank correlation coefficient test to assess for association between post-operative whole-blood thiamine levels and post-operative whole-blood lactate levels. A *p* value of 0.05 was used to determine statistical significance for all analyses.

## Results

From May 2018 to October 2018, a total of 82 patients were recruited, with 2 excluded after enrollment due to surgery occurring without CPB, and 2 excluded due to 24-h delay in measurement of postoperative TDP level. Data was available for analysis in 78 patients.

The characteristics of the patients at baseline are collated into their demographic data (Tables 1, 2) and their operation type (Table 3).

**Table 1:** Baseline patient characteristics

Demographic variable	Mean (SD)	Range
Age (years)	61.53 (11.55)	30–84
Creatinine (µmol/L)	83.5 (27.83)	46–256
Haemoglobin (mg/L)	137.9 (16.2)	87–169
Height (cm)	171.2 (8.9)	157–190
Weight (kg)	84.8 (15.1)	55–117
Body Mass Index (kg/m^2^)	28.9 (4.8)	20–45
CPB time (min)	108.5 (47.3)	42–299
Cross-clamp time (min)	81.9 (35.1)	28–204

SD, standard deviation

**Table 2:** Baseline patient characteristics 2

Demographic variable	Number (%)
Gender	
Male	24 (30.8)
Female	54 (69.2)
Diabetes mellitus	
Yes	26 (33.3)
No	52 (66.7)

**Table 3:** Type of operation conducted

Type of surgery	Number (%)
CABG	45 (57.7)
CABG and valve replacement/repair	6 (7.7)
Valve replacement/repair	26 (33.3)
Other (atrial septal defect repair)	1 (1.3)

CABG, coronary artery bypass graft

**Fig. 2:**
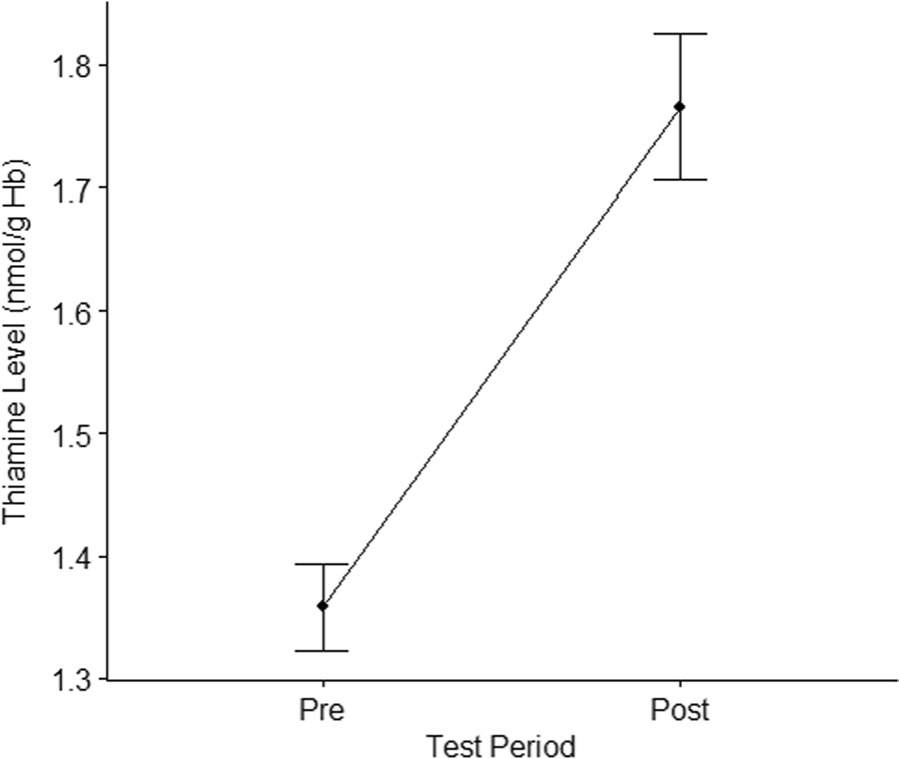
Mean thiamine levels pre- and post-cardiopulmonary bypass. nmol/g Hb, nanomoles per gram of haemoglobin

Mean post-CPB thiamine levels were 1.77 nmol/g Hb (standard deviation [SD] 0.53, 95% confidence interval [CI] 1.43–1.88), significantly higher than the mean pre-CPB level 1.36 nmol/g Hb (SD 0.31, 95% CI 1.29–1.43, *p* < 0.001) (Fig. 2).

There were six patients found to be thiamine deficient pre-CPB, as defined by the laboratory reference range of < 0.90 nmol/g Hb (Table 4). All six patients had post-CPB thiamine levels that had increased to within normal range. A total of four patients had post-CPB thiamine levels that were lower than their pre-CPB level, although only one patient’s thiamine level was below the normal reference range (Table 5). None of these four patients had thiamine levels lower than the normal laboratory reference range pre-CPB.

**Table 4:** Patients with thiamine levels below the normal laboratory reference range

Patient No	Pre-CPB thiamine level (nmol/g Hb)	Post-CPB thiamine level (nmol/g Hb)
2	0.69	1.59
31	0.87	0.98
37	0.82	0.92
64	0.84	1.13
72	0.85	0.95
77	0.84	1.49

CPB, cardiopulmonary bypass

**Table 5:** Patients with decreased thiamine levels post-cardiopulmonary bypass

Patient No	Pre-CPB thiamine level (nmol/g Hb)	Post-CPB thiamine level (nmol/g Hb)
8	1.85	1.69
15	1.68	0.86
46	2.11	1.76
69	1.49	1.48

CPB, cardiopulmonary bypass

**Fig. 3:**
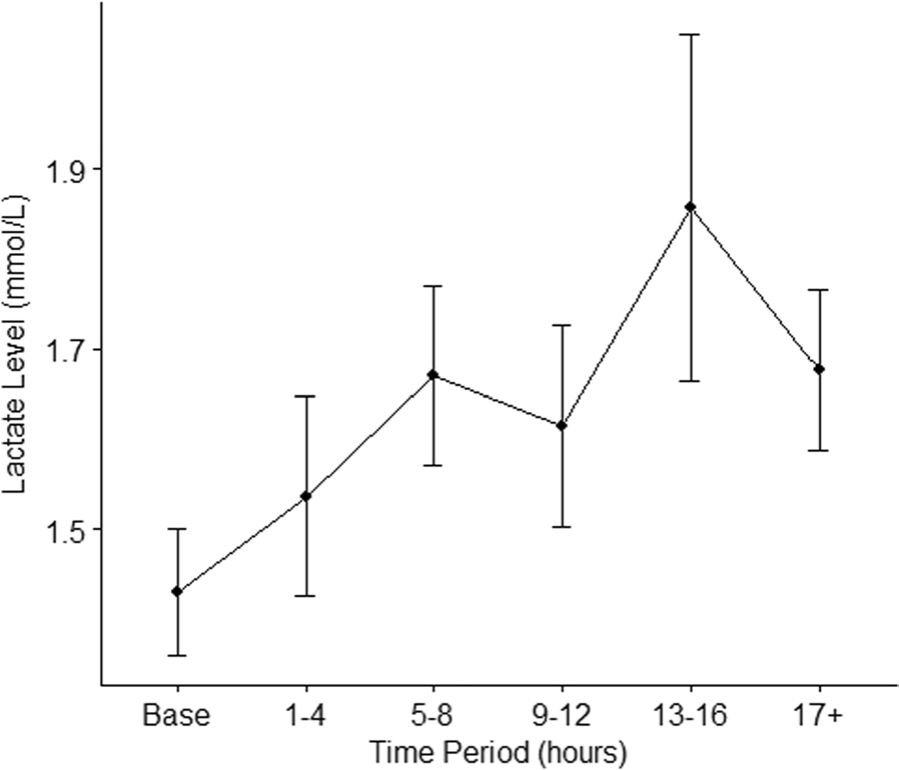
Mean lactate levels and confidence intervals during the first 24 h post-cardiopulmonary bypass. mmol/L, milimoles per litre

Post-operative whole-blood lactate levels were recorded at baseline and collated into five time-groups due to slight variations around sample collection times: 1–4 h, 5–8 h, 9–12 h, 13–16 h and 17 + hours. Analysis by Kruskal–Wallis tests between each time-group compared to initial mean whole-blood lactate levels showed a significant difference between baseline and 13–16 h (*p* < 0.05), indicating lactate levels were significantly higher at 13–16 h than baseline. Spearman’s rank correlation coefficient analysis found no significant correlation between whole-blood thiamine and lactate levels (Table 6). A trend was observed in rising lactate levels across the time groups, however this was not statistically significant (p > 0.05) and all mean values remained within normal reference range (< 2 mmol/L) (Fig. 3, Table 4).

**Table 6:** Correlation analysis of post-operative whole-blood thiamine levels and lactate levels

Correlations																	
	Post-Thiamine	Lactate	Lactate	Lactate	Lactate	Lactate	Lactate	Lactate	Lactate	Lactate	Lactate	Lactate	Lactate	Lactate	Lactate	Lactate	Lactate
Correlation coefficient	1.000	.175	.111	.072	.012	.087	.205	.295	.164	.080	.112	.317	.452	.600	.200	− 1.000	− 1.000
*p* value (2-tailed)	–	.122	.326	.524	.913	.451	.091	.032	.338	.718	.703	.406	.260	.285	.800	–	–
N	80	80	80	80	80	77	69	53	36	23	14	9	8	5	4	2	2

## Discussion

This study demonstrated an unexpected relationship between thiamine levels and CPB. To our knowledge, only two previous studies have measured thiamine levels pre- and post-CPB [10, 11]. Both studies analysed smaller sample sizes and found that thiamine levels decreased following CPB. This study has demonstrated the opposite, with a significant increase in post-CPB thiamine levels.

There are methodological differences between this study and previous research that may account for the opposing results. Firstly, differing study populations where Donnino et al. exclusively evaluated patients undergoing CABG, whereas this study analysed data from a variety of cardiothoracic surgical procedures provided the patient underwent CPB [10]. Crucially, different measurement techniques were utilized, where Donnino et al. and Anderson et al. both centrifuged EDTA samples and measured plasma thiamine levels and presented results as a nmol/L [10, 11]. In contrast, this study tested whole-blood thiamine levels and calculated a thiamine-haemoglobin ratio for each patient, presented in nmol/g Hb. Given the majority of thiamine is found in red blood cells with minimal present in plasma, the measurement of whole-blood thiamine and calculating a thiamine-haemoglobin ratio would produce different results compared to plasma thiamine measurements [17]. The measurement of whole-blood thiamine is a more reliable measure of thiamine deficiency, given that plasma thiamine levels are subject to influence by recent dietary intake [17].

Methodological differences aside, the reason for the increase in post-CPB whole-blood thiamine levels is not immediately apparent. The biochemistry and metabolism of thiamine is the subject of ongoing research, particularly as it relates to critical illness [18]. Approximately 30 mg of thiamine can be stored in an average adult and this rapidly depletes without ongoing intake [18]. Thiamine utilization appears to increase during physiological and metabolic stress, whereby consumption causes a decrease in measured levels, and this may be the result of mitochondrial activity and oxidative stress [11, 19].

It is routine practice at TUH to induce hypothermia of 32–34 °C during CPB, aiming to reduce metabolic demand to protect the patients’ organs from ischaemic injury. The post-CPB thiamine levels were taken immediately post-surgery on arrival to ICU, during which time patients are often still re-warming. It may be that whole-blood thiamine levels were artificially high due to a period of hypothermia-induced low pyruvate dehydrogenase (PDH) enzyme activity, and the reduced metabolic demand limited a significant rise in lactate [11]. Donnino et al. and Anderson et al. may not have observed this if their patients were already re-warmed when the first post-CPB thiamine level was taken [10, 11]. Reduced PDH activity may occur during CPB unrelated to hypothermia, and this may be an alternative explanation for the increased thiamine levels post-CPB recorded in this study [12]. The reduced PDH activity would decrease thiamine utilization during CPB, causing increased thiamine levels post-CPB.

Although not statistically significant, this study showed a trend of increasing post-operative whole-blood lactate levels since time of operation. While lactate levels remained within normal range during the period of data collection, this rise may correlate with enzyme reactivation and increased metabolic demand with return to normal systemic temperatures, and thus an increased thiamine demand [2, 10–12]. Thiamine levels measured during this time may have decreased from baseline, which may account for the observations by Donnino et al. and this could be investigated further in future research [10]. As the majority of the study cohort had normal thiamine levels post-CPB, this could account for the observation that the post-operative whole-blood lactate levels remained within normal limits in the study population. No correlation between post-operative thiamine and lactate levels was identified, which opposed the previous finding by Anderson et al. [11].

It is also noteworthy that there was a low incidence of pre-CPB thiamine deficiency within the study cohort, as defined by the laboratory reference range. Thiamine levels had risen to within normal range for all of these patients on their post-CPB testing. This makes little physiological sense but may be explained by the ‘use and demand’ hypothesis previously discussed.

Based on the apparent CPB-related thiamine depletion demonstrated in previous research, numerous RCTs have been undertaken whereby thiamine is administered to patients undergoing CPB [10–14]. No RCT has shown that thiamine supplementation has significant effect on post-operative lactate levels or other outcome measures, though studies have been under-powered to detect such effects [12–14]. If nothing else, the results of this study highlight the need for careful re-consideration of the use of resources regarding future research into this area.

However, some questions remain unanswered. An interesting phenomenon was observed in Anderson et al. whereby patients who received thiamine supplementation had reduced PDH activity [12]. Lomivorotov et al. recently performed a pilot feasibility trial of thiamine supplementation in patients undergoing CPB and noted a trend toward higher lactate levels in the first 24 h post-operatively in those who received thiamine supplementation [14]. Biological plausibility would support that reduced PDH activity would correlate with higher lactate levels, but the mechanism by which thiamine supplementation would be associated with reduced PDH activity is uncertain.

Also of unclear pathophysiology is that upon serial measurements of serum thiamine levels, Anderson et al. found that the number of patients who were thiamine deficient increased with time post-operatively, though the absolute numbers were small [12]. Donnino et al. showed that within a sepsis cohort, the subgroup of patients who were thiamine deficient and received thiamine supplementation had lower lactate levels and a trend towards reduced mortality [20]. Perhaps future research should focus specifically on the subset of patients with established pre-operative thiamine deficiency.

### Limitations

While this study is the largest known undertaken to measure pre- and post-CPB thiamine levels, there are several limitations. This study only performed a single post-operative measurement of whole-blood thiamine levels, immediately on admission to ICU. This was based on the hypothesis that the causative factor for thiamine depletion would be related to the process of undergoing cardiothoracic surgery and CPB, and previous research that demonstrated a significant reduction in thiamine levels immediately post-operatively [10, 11]. However, given prior research showed sustained thiamine depletion at 6-h and 24-h post-operatively, perhaps serial whole-blood thiamine measurements at later time points may have yielded differing results [10, 11]. Additionally, this study did not record the amount of blood transfusion received by each patient, which may have influenced their whole-body thiamine levels based on the current understanding of thiamine physiology.

Lactate is subject to influence from a wide variety of variables that impact oxygen delivery and utilisation, for example, vasopressor choice and dose, and cardiac output. This study collected minimal data on these potential influences, and these may have provided further enlightenment to the obtained results. It also did not record the patient’s admission temperature to ICU post-operatively, which may have correlated to the level of PDH activity and hence, both whole-blood thiamine and lactate levels.

## Conclusion

In this study of elective surgical patients undergoing CPB, blood thiamine levels were found to increase immediately following CPB. There was no significant relationship observed between thiamine levels and post-operative lactate levels.

## Data Availability

The datasets used and/or analysed during the current study are available from the corresponding author.

## Abbreviations

CPB: Cardiopulmonary bypass
SD: Standard deviation
CI: Confidence interval
TDP: Thiamine diphosphate
PDH: Pyruvate dehydrogenase
RCT: Randomized control trial
ICU: Intensive care unit
BMI: Body mass index
EDTA: Ethylene-diamine-tetraacetic acid
Hb: Haemoglobin
HPLC: High performance liquid chromatography
nmol/L: Nanomoles per litre
nmol/g Hb: Nanomoles per gram of haemoglobin
CABG: Coronary artery bypass graft
